# Variations in Schedule III prescription patterns in a Medicaid population pre- and post-policy

**DOI:** 10.1038/s41598-021-86409-6

**Published:** 2021-03-30

**Authors:** Radhakrishnan Nagarajan, Jeffery Talbert, Craig S. Miller, Jeffrey Ebersole

**Affiliations:** 1 Center for Oral and Systemic Health, Marshfield Clinic Research Institute, Marshfield Clinic Health System, Marshfield, WI USA; 2 Department of Pharmacy Practice and Science, College of Pharmacy, University of Kentucky, Lexington, KY USA; 3 Department of Oral Health Practice, College of Dentistry, University of Kentucky, Lexington, KY USA; 4 School of Dental Medicine, University of Nevada Las Vegas, Las Vegas, NV USA

**Keywords:** Scientific data, Health care, Dentistry, Health policy

## Abstract

The present study investigated variations in patient movement patterns between prescribers before and after House Bill 1 (HB1) implementation in Kentucky using network abstractions (PPN: prescriber-prescriber networks) from a one-month cross-sectional Schedule III prescription data in a Medicaid population. Network characteristics such as degree centrality distribution of PPN was positively skewed and revealed Dental Practitioners to be the highly connected specialty with opioid analgesic hydrocodone-acetaminophen to be the most commonly prescribed drug. Taxonomy enrichment of the prescriber specialties in PPN using chi-square test revealed a reduction in the enriched taxonomies Post-HB1 compared to Pre-HB1 with Dental practitioners being constitutively enriched (*p* < 0.05). PPNs were also found to exhibit rich community structure revealing inherent clustering of prescribers as a result of patient movement, and were markedly different from those generated by random graph models. The magnitude of deviation from random graphs decreased Post-HB1 relative to Pre-HB1. The proposed network approach provides system-level insights into prescribers with potential to complement classical reductionist approaches and aggregate statistical measures used in assessing changes in prescription patterns pre- and post- policy implementation. It can provide preliminary cues into drug seeking behavior, and facilitate targeted surveillance of prescriber communities.

## Introduction

The Centers for Disease Control and Prevention (CDC) provided compelling evidence of increasing trends (2009–2017) in drug overdose deaths including those from prescription opioids (CDC Wide-ranging Online Data for Epidemiologic Research, WONDER Online Database, 2018)^1^. CDC WONDER also reported deaths from prescription opioids (17,029) to be higher compared to that from heroin (15,482), cocaine (13,942), benzodiazepines (11,537), psychostimulants (10,333) and antidepressants (5269) in 2017. Non-medical use of opioid analgesics has especially gained attention when it comes to overdose deaths^2^. Non-medical use^3^ can be stratified under five broad categories (misuse, abuse, addiction, physical dependence and tolerance)^4^. Prescription drugs fall under the schedules (I, II, III, IV) with varying physical and psychological dependence (https://www.dea.gov/drug-scheduling). A recent study reported considerable misuse of prescription opioids in a large population^5^ with a majority of the misuse attributed to relieving pain. Pain relievers such as hydrocodone-acetaminophen are also one of the commonly prescribed medications by dental practitioners^6^ during the acute post-operative period, after invasive dental procedures^7^. Opioid analgesic hydrocodone-acetaminophen was initially classified under Schedule III but were later rescheduled under Schedule II/IIN analgesic opioids by the Drug Enforcement Association (Oct. 6, 2014) with increased regulatory, criminal and civil sanctions. While recent studies have reported non-medical use of pain medications in dental settings from patient self-reported questionnaire^8^, others have demonstrated increasing trends (2010–2015) in opioid prescribing by dental practitioners from commercial claims data^9^. The American Dental Association official statement in response to the opioid crisis recommended a limit on opioid usage with a duration of seven days for treating acute pain in an effort to reduce overprescribing practices. The newer guidelines suggest shorter limits on prescribing duration.

Classical approaches for investigating prescription patterns have focused traditionally on aggregate statistical measures with emphasis on the number of prescriptions dispensed and temporal trends across specific drugs, drug class, demographics, prescriber specialties and policy implementation^10–12^. However, increasing evidence of patients seeking drugs across multiple prescribers for non-medical use (e.g. doctor-shopping, provider shopping, drug diversion)^13^ demands developing novel approaches that can complement traditional characterization by incorporating patient-movement patterns. More specifically, such an understanding has the potential to provide system-level insights into the concerted behavior of prescribers that may not be readily apparent from a reductionist representation where patients and prescribers are treated as independent entities^14^. As shown in the present study, it can also provide insights into prescriber communities that represent clustering of prescribers into groups and quantify its variation pre- and post-intervention.

Kentucky was ranked at number eight in prescription drug overdose mortality with 27.9 deaths per 100,000 in 2017 and had at that time seen an increasing prescription drug abuse problem for several years (2017 Opioid Summaries from drugabuse.gov). The state was an early adopter of electronic prescription drug monitoring program (PDMP). PDMPs have been implemented across 49 states (pdmpassist.org) for combating prescription drug (Schedule II–IV) abuse and diversion. Kentucky passed House Bill 1 (**HB1**) in April, 2012 requiring mandatory use of the prescription drug monitoring program (KASPER: Kentucky All Schedule Prescription Electronic Reporting) for prescribing controlled substances. A recent scoping review summarized the impact of PDMPs across diverse settings including opioid misuse, mortality, and across dental urgent care centers^15^.

The present study investigated Schedule III prescription patterns from Medicaid claims data from Kentucky in a single month before HB1 (September, 2011, **Pre-HB1**) and after HB1 (September 2012, **Post-HB1**) implementation with a focus on Medicaid population using traditional aggregate measures as well as network abstractions (**PPN**: prescriber-prescriber network)^14^. The results presented demonstrate a marked decrease Post-HB1 relative to Pre-HB1 in aggregate measures such as prescription counts. PPN characteristics including degree centrality distributions, taxonomy enrichment and strength of the community structure using random graphs as internal controls were also shown to exhibit marked variations between Pre-HB1 and Post-HB1. These results elucidate the usefulness of network analytics approaches in complementing more traditional characterization of prescription patterns and their potential to serve as evaluation tools, visualizing patient movement patterns and assessing changes pre- and post-policy implementation.

## Materials and methods

### Data description and implementation

The data investigated in the present study are outpatient pharmaceutical claims data retrieved from Kentucky Medicaid Services consisting of encrypted prescriber IDs (National Provider Identifier), encrypted patient IDs, National Drug Code (NDC) description of Schedule III drugs, and GIS locations of the prescribers and specialty of the prescribers in the month of September in 2011 (Pre-HB1) and 2012 (Post-HB1). The time period is restricted to the same month across Pre-HB1 and Post-HB1 in order to minimize the effect of potential confounders (e.g. seasonal effects). The time period also was restricted to a single month (30-day period) to focus on a snapshot of typical prescribing patterns in a controlled time frame with minimal likelihood of patients seeking multiple prescriptions of Schedule III drugs. The study was approved by the University of Kentucky Institutional Review Board and the data can be requested from the Cabinet for Health and Family Services (https://chfs.ky.gov/agencies/ohda/Pages/default.aspx). Prescribers were classified under their respective broad specialties using the first four letters of the 10 digit Taxonomy Code provided by Medicaid and the drug names were identified from their NDC description. The taxonomy codes of General Practice Dentists, Endodontist, Prosthodontist, Oral & Maxillofacial Surgeons and Pediatric Dentists were merged under the common term “Dental Practitioners” with taxonomy code (1223). The taxonomy codes were not available for 126 prescribers in 2011 and 86 prescribers in 2012, therefore they were eliminated from further consideration. All the approaches in the present study were implemented in open-source environment R using publicly available R libraries such as (igraph, linkcomm)^16,17^ for network analysis and community structure detection. Open-source package Gephi^18^ was used to generate geo-spatial visualization of the PPNs.

### Demographics

The mean and standard deviation of patient’s age who were prescribed Schedule III prescriptions were 39.1 ± 14.6 years (Pre-HB1) and 41.3 ± 13.9 years (Post-HB1) respectively. Their corresponding sex ratios (males/females) were 46% Pre-HB1 and 48% Post-HB1. The mean and standard deviation of patient’s age who were prescribed Schedule III prescriptions by dentists were 28.7.1 ± 11.7 years (Pre-HB1) and 30.0 ± 11.9 years (Post-HB1) respectively. Their corresponding sex ratios were 43% Pre-HB1 and 40% Post-HB1.

### PPN construction

**Figure 1:**
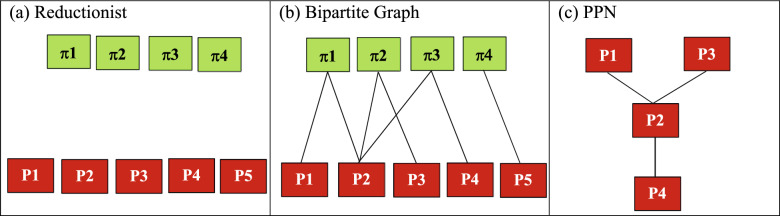
Reductionist representation of patients (π1, π2, π3, π4, green squares) and prescribers (P1, P2, P3, P4, P5, red squares) as independent entities is shown in (**a**). System-level abstraction of patient movement between prescribers using a bipartite graph is shown in (**b**). Prescriber-Prescriber Network (PPN) obtained from the unipartite projection of the bipartite graph in (**b**), representing the association between prescribers as a result of patient movement is shown in (**c**). (Figures were generated using Microsoft Word, https://www.microsoft.com/en-us/).

Classical reductionist representation that treat the patients and prescribers as independent entities is shown in Fig. 1a. It is important to note that the patients and prescribers are considered as independent entities in the reductionist representation. System-level representation of the prescribers as a result of patient movement between them using bipartite graphs extending this example is shown in Fig. 1b^14^. An edge from a patient to a prescriber represents a patient who was prescribed Schedule III prescription by that prescriber. Multiple prescriptions from distinct prescribers are represented by multiple edges (e.g. π2 − P2, π2 − P3), Fig. 1b. While the bipartite graph in Fig. 1b, represents the association between patients and prescribers, a unipartite projection can be generated representing the association solely between the prescribers (PPN) as shown in Fig. 1c. For instance, since patient π2 is prescribed Schedule III drugs by P2 and P3, these prescribers are connected by an edge in Fig. 1c. Prescriber P5 is a singleton node with zero patients moving between P5 and other prescribers, hence excluded from the PPN. Since there is no directionality of the edges, PPNs by very construction are undirected graphs. Degree centrality prescribers (P1, P2, P3, P4) in Fig. 1c is (1, 3, 1, 1) respectively. It is important to note that in the absence of patient movement between multiple prescribers, the PPN in Fig. 1c would essentially be devoid of any edges providing no distinct advantage over the reductionist representation in Fig. 1a.

### Degree centrality distribution

Degree centrality^19^ of a provider in PPN is given by the number of distinct providers that the given provider is connected to as a result of patient movement. Degree centrality distribution has the potential to provide novel insights into the PPN topology^20^. Of interest is to note that targeted intervention of prescribers with high-degree centrality from the network can considerably fragment the network and in turn impact patient movement patterns. For instance, targeted removal of P2 in Fig. 1c renders P1, P3 and P4 as isolated nodes. The present study investigated highly connected specialties from their degree centrality in the PPN and variations in degree centrality distribution Pre-HB1 and Post-HB1. Wilcoxon-rank-sum test was used to investigate statistically significant (α = 0.05) variations in degree centrality distributions Pre-HB1 and Post-HB1.

### Taxonomy enrichment

Taxonomy enrichment was used to investigate statistically significant proportion of a given prescriber specialty or taxonomy in the given PPN. Enrichment of a prescriber specialty or taxonomy (*T*) in the PPN was determined from a 2 × 2 contingency table in conjunction with chi-square test (α = 0.05). The elements of the 2 × 2 table comprised of four categories corresponding to (1) number of unique prescribers with taxonomy *T* in PPN, (2) number of unique prescribers with taxonomy *T* not in PPN, (3) number of unique prescribers with other taxonomies in PPN, and (4) number of unique prescribers with other taxonomies not in PPN. Since the above procedure involves multiple testing, Bonferroni correction was used to control for family-wise error rate with the corrected significance level given by (α* = α/n) where n represented the total number of tests.

### Community structure and surrogate testing

Communities in a given network represents a natural clustering of that network where some subsets of nodes are more densely connected than the rest. Several community structure detection algorithms have been proposed in the past^21,22^. The choice of these algorithms is often context-specific. The present study investigated community structure using an overlapping community structure detection algorithm^16,22^ that permits a prescriber to be a member of more than one community. Encouraged by our recent findings^14^, random graph surrogates^23^ were used to investigate whether the PPN communities were different from those that can be generated from random graph models that retain the degree distribution. Absence of significant differences between the PPN properties and those of their random graph surrogates would essentially imply that random graphs can be used as a generative model of PPN. In case of marked differences between random graphs and the PPN, random graphs can serve as useful controls in quantifying the difference. This is especially helpful since random graphs are constrained realizations and retain the nodes and edges of the PPN Pre-HB1 and Post-HB1 that may not be preserved in a direct comparison. Subsequently, the magnitude of deviation of the community structure in the given PPN from those of their random graph surrogate counterpart was quantified by their S estimates^24^, given by $\mathrm{S}=\frac{|{m}_{o}-{\mu }_{surr}|}{{\sigma }_{surr}}$, where ${m}_{o}$ represent the maximum partition density estimate of the PPN and $({\mu }_{surr}{,\sigma }_{surr})$ represent the mean and standard deviation of the maximum partition density estimates of ${n}_{s}=99$ random graph surrogate counterparts with S estimates deemed as significant when S > 2 ^24^.

## Results

### Prescription counts and PPN visualization

**Figure 2:**
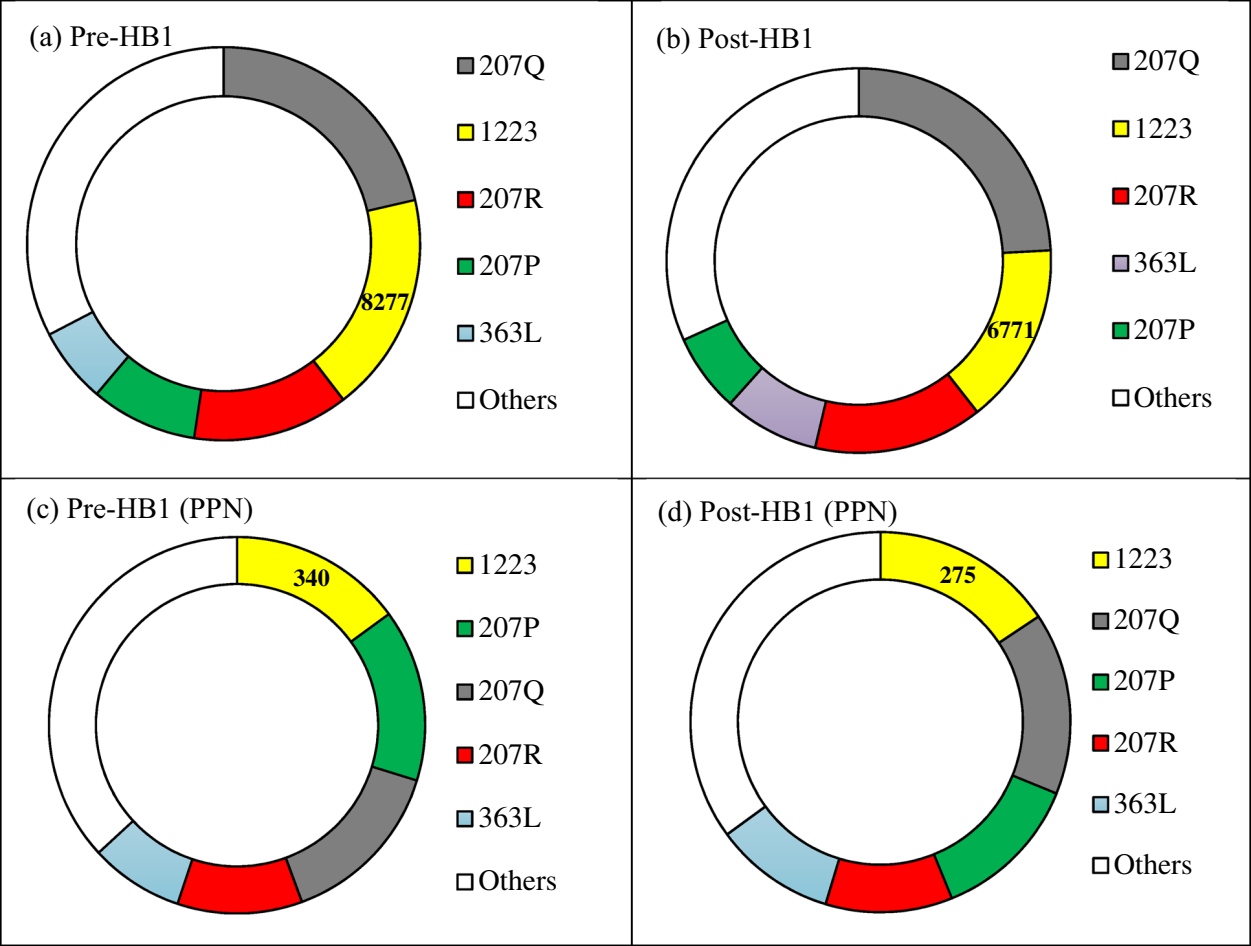
Top five prescriber specialties ranked by the total number of prescriptions including duplicate prescriptions Pre-HB1 and Post-HB1 is shown in (**a**) and (**b**), respectively. Top five prescriber specialties ranked in the PPNs at Pre-HB1 and Post-HB1 is shown in (**c**) and (**d**) respectively. The specialties corresponding to the taxonomy codes in each of the subplots were (207Q: Family Medicine; 1223: Dental Practitioners; 207R: Internal Medicine; 207P: Emergency Medicine; 363L: Nurse Practitioner) with Dental Practitioners shown in yellow and a generic category that includes all the remaining specialty in white in each of the subplots. (Figures were generated using Microsoft Word, https://www.microsoft.com/en-us/).

**Figure 3:**
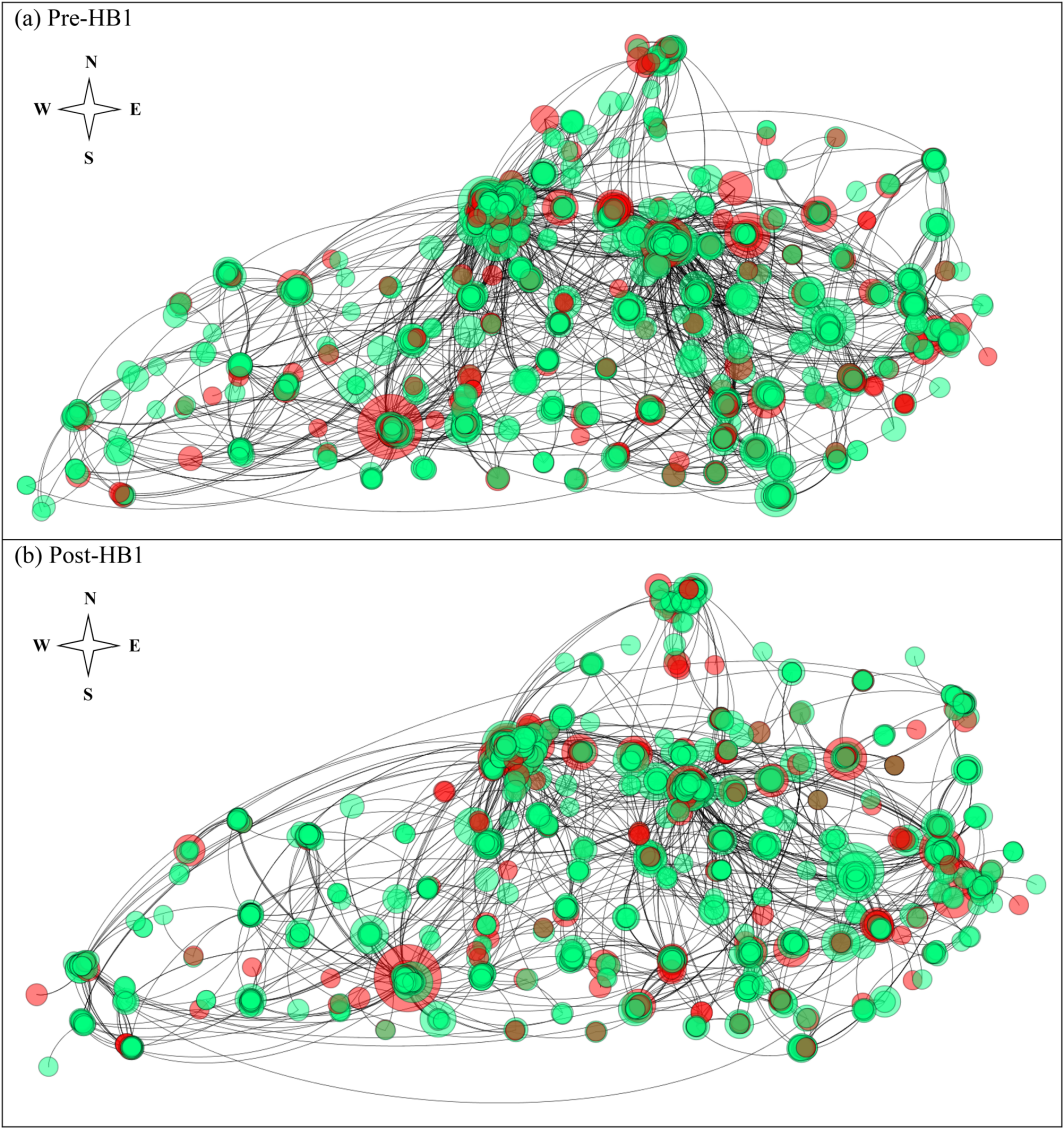
Geospatial layout of the prescribers of Schedule III drugs comprising of Dental Practitioners (red circles) and other specialties (green circles) in Kentucky Pre-HB1 and Post-HB1 is shown in (**a**) and (**b**) respectively. Size of a node in the above figure is proportional to its degree centrality with nodes stacked behind one another in dense areas. (Figures were generated using Gephi, https://gephi.org/).

Total number of Schedule III prescriptions including duplicate prescriptions dispensed decreased by 2.9% from Pre-HB1 (45,566) to Post-HB1 (44,285). Top five specialties ranked by the total number of prescriptions dispensed Pre-HB1 and Post-HB1 were Family Medicine, Dental Practitioners, Internal Medicine, Emergency Medicine, Nurse Practitioner, Fig. 2a,b. More specifically, Dental Practitioners was ranked as the second highest prescriber of Schedule III prescriptions after Family Medicine Pre-HB1 and Post-HB1 and the total number of Schedule III prescriptions by dental practitioners showed a marked decrease Post-HB1 (~ 18%) relative to Pre-HB1 and consisted primarily of hydrocodone-acetaminophen combinations. However, the highest prescriber specialty in the PPN reflected by the degree centrality was Dental Practitioners, Fig. 2c,d. As noted earlier, Fig. 1c, the edges in the PPN represents the patients who obtained multiple prescriptions from distinct prescribers including those with the same specialty. The top five prescriber specialties connected to Dental practitioners in the PPN ranked by the number of edges Pre-HB1 were (Dental Practitioners, Emergency Medicine, Family Medicine, Internal Medicine and Nurse Practitioner) and Post-HB1 were (Dental practitioners, Family Medicine, Internal Medicine, Emergency Medicine and Nurse Practitioner). Geo-spatial layout of PPNs corresponding to Pre-HB1 and Post-HB1 generated using Gephi^18^ is shown in Fig. 3a,b respectively. The coordinates for a small proportion of the prescribers (~ 3.3%) and (~ 3.6%) were unknown for Pre-HB1 and Post-HB1, hence not shown in Fig. 3. The Geo-spatial layout revealed patients traveling across considerable distances within the state to obtain Schedule III prescriptions from multiple prescribers within a single month and a marked reduction in the number of nodes as well as edges Post-HB1 (nodes = 1696, edges = 1561) relative to Pre-HB1 (nodes = 2200, edges = 2533), Fig. 3 indicating a decrease in the number of prescribers in the PPN as well as patients who were prescribed Schedule III drugs by multiple prescribers.

### PPN degree distribution and taxonomy enrichment

**Figure 4:**
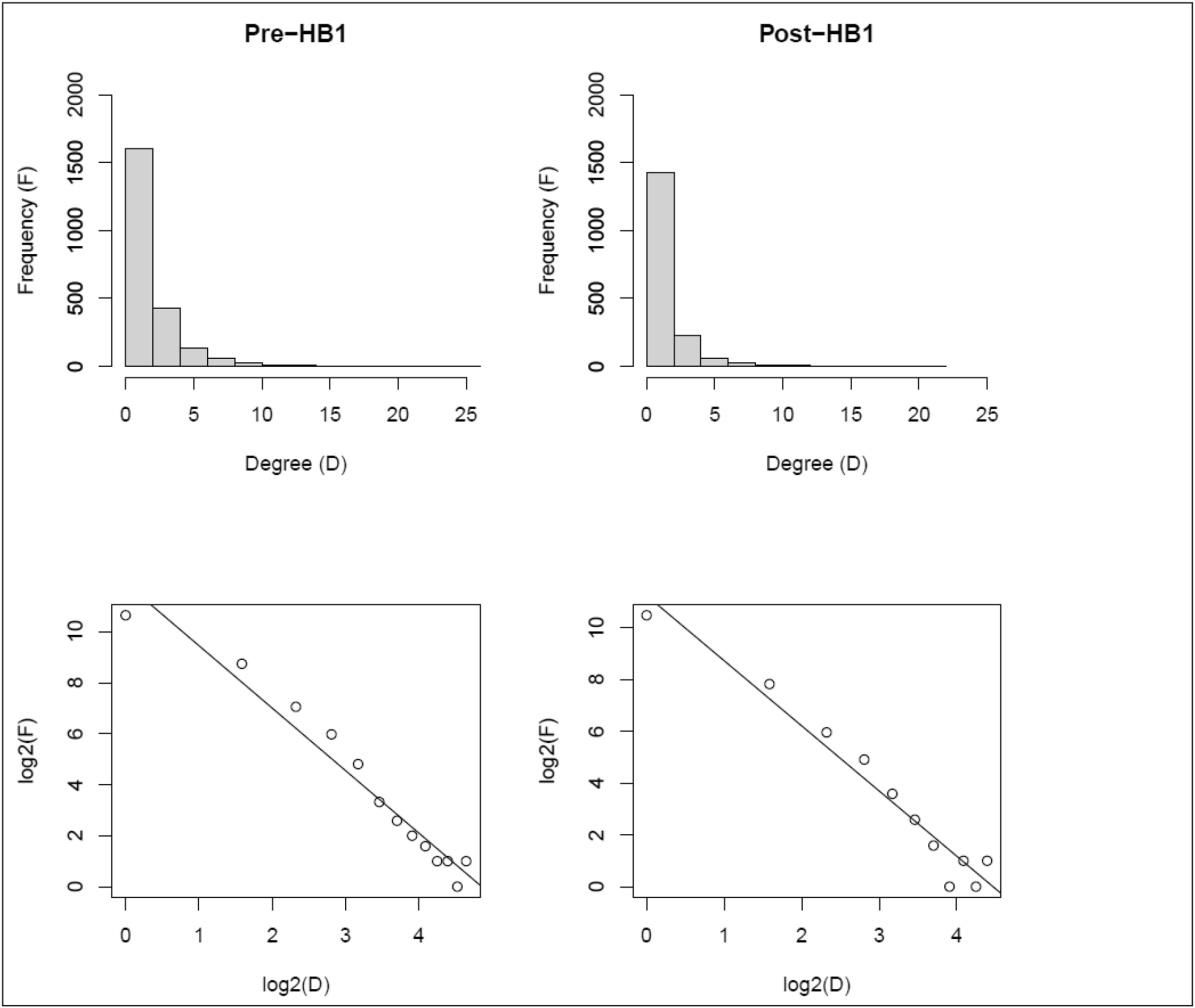
Degree centrality distributions of prescribers in the PPN Pre-HB1 and Post-HB1 are shown in the left and right columns respectively. A linear fit to the degree centrality distribution in the log–log scale is shown right below the respective plots. Figures were generated using R, https://www.r-project.org/.

The degree centrality distributions of the PPN was positively skewed Pre-HB1 (skewness = 3.3, kurtosis = 17.9) and Post-HB1 (skewness = 3.7, kurtosis = 22.6) indicating a small proportion of highly connected prescribers, Fig. 4 (top). A linear fit of the fraction of the nodes $P\left(k\right)$ with degree centrality $k$ in the log–log scale were indicative of potential power-law behavior^19,20^ $P(k)={k}^{-\gamma }$, with exponents $\gamma =2.45 (p<0.05)$ and $\gamma =2.51 (p<0.05)$ at Pre-HB1 and Post-HB1 respectively, Fig. 4 (bottom). Changes in properties of PPN degree distribution should not be surprising and may be an outcome of “chilling effect”^25^ that often follow policy implementations. Power-law distributions can exhibit interesting characteristics such as scale-free behavior. Power-law distributions are scale-invariant as scaling the degree $k$ by a constant factor $\delta$ does not change the underlying properties of the network i.e. $P\left(\delta k\right)={\delta }^{-\gamma }\cdot P(k)$. This behavior has also been attributed to universality where properties of networks are independent of their size. While these results might provide preliminary cues, a more rigorous investigation may be required to claim power-law behavior of PPN^26,27^. In the present study, ranking the prescribers by their degree centrality in the PPN revealed Dental Practitioners to be consistently ranked as the most connected specialty in the PPN. More importantly, the number of dental practitioners in the PPN decreased by ~ 19% and their edges decreased by ~ 30% Post-HB1 relative to Pre-HB1. The degree centrality distributions of the entire PPN and those of dental practitioners were statistically different between Pre-HB1 and Post-HB1 as revealed by Wilcoxon rank-sum test (*p* < 0.05). Taxonomy enrichment of the Top 5 providers, Fig. 2, in the PPN using chi-square test (α = 0.05) revealed specialties (Dental Practitioners, Emergency Medicine, Family Medicine, Internal Medicine) to be enriched Pre-HB1 and (Dental Practitioners, Emergency Medicine, Internal Medicine) to be enriched Post-HB1. More importantly, Family Medicine which was one of the highest prescribers of Schedule III drugs Pre-HB1 was not enriched Post-HB1 in the PPN.

### PPN community structure

Rather than compare the strengths of the communities Pre-HB1 and Post-HB1 directly, the present study used random graph surrogates as internal controls. Surrogate testing of the Schedule III PPN largest connected component resulted in S values (S = 82.6 ≫ 2, Pre-HB1) and (S = 48.8 ≫ 2, Post-HB1) rejecting random graph models as potential generative mechanisms of the community structure in the PPN. More importantly, the deviation from the random graph surrogates reflected by the magnitude of the S values, exhibited a marked decrease from Pre-HB1 to Post-HB1.

## Discussion

Prescription drug abuse is a major public health concern. A majority of existing approaches have focused on understanding prescription counts across specific drugs, prescriber specialties and their temporal trends using a variety of data sources. While helpful, these studies have been reductionist in nature and treat prescribers and patients as independent entities. Recent studies have provided compelling evidence of patients seeking drugs from multiple prescribers for non-medical purposes. These in turn demand novel approaches that rely on network analytics for understanding patient movement patterns across prescribers. Such a system-level understanding can assist in identifying prescriber communities and specialties for targeted surveillance. Understanding the variations in the properties of these networks can also serve as useful tools in assessing the impact of policies.

Results in this study revealed a marked decrease in the number of Schedule III prescriptions Post-HB1 relative to Pre-HB1 in the Kentucky Medicaid population, with certain prescriber specialties such as Dental Practitioners to be highly ranked Pre-HB1 as well as Post-HB1. As noted earlier, changes in prescription counts and distributional signatures of PPN Post-HB1 relative to Pre-HB1 may be an outcome of “chilling effect”^25^ that usually accompany policy implementations. PPN visualization revealed patient-movement patterns across multiple prescribers over considerable geographical distances that decreased Post-HB1 relative to Pre-HB1. While it is uncommon for patients to seek the same schedule prescription drug across multiple prescribers within a single month time frame, it is possible that this may include patients having multiple asynchronous and chronic health conditions. Findings from the present study such as the skewed degree centrality distributions in the PPN indicate the critical role of a small number of prescribers and prescriber specialties such as dental practitioners with very high connectivity in the PPN comprising the tail of the distribution. Skewed degree centrality distributions such as power-law distributions have been attributed to several interesting phenomena including robustness to random attacks or random interventions^28^. Preliminary investigation revealed a linear trend in the log–log plot of PPN degree centrality distribution. Since a large number of prescribers are not highly connected in the PPN, random attacks of the PPN nodes are likely to have negligible impact whereas targeted intervention and surveillance of the highly connected prescribers and select prescriber communities and specialties may have profound impact on the patient movement patterns including significant fragmentation of these networks. Thus, understanding the properties of PPN has the potential to assist in developing targeted surveillance strategies. In the present study, Dental Practitioners had the highest degree centrality in the PPN, indicating that patients who were prescribed Schedule III drugs by Dental Practitioners were also prescribed Schedule III drugs by other Dental Practitioners and other prescriber specialties. Taxonomy enrichment also revealed Dental Practitioners to be enriched in the PPN Pre-HB1 and Post-HB1 constitutively with hydrocodone-acetaminophen to be the most prescribed Schedule III opioid analgesic.

The present study also investigated the presence of community structure in the connected component of the PPN where it is possible to traverse from a given node to any other node in the PPN. Presence of communities essentially reflect inherent clustering in the PPN where sub-groups of prescribers and prescriber specialties are more densely connected to one another as opposed to the rest. Such an understanding can again assist in targeted surveillance of prescriber communities. This was accomplished using overlapping community structure detection algorithms and random graph surrogate models. Overlapping community structure detection is well suited for the present study as it permits a prescriber to be a member of more than one community as a result of patient movement between distinct prescriber specialties. Community structure in the given PPN was compared to those that can be generated using random graph models that retain the degree centrality distribution of the PPN. Rationale behind such a comparison are two folds: (1) determine whether in-silico random graph models can prove to be useful in generating community characteristics of the PPN and (2) assess the deviation of the properties of the PPN from those of random models using these models as internal controls since the random graphs retain the nodes, edges and degree centrality distribution of the PPN. The surrogates also implicitly retain characteristics such as network density given by the ratio of actual edges (*e*) divided by the number of possible connections given by 2*e*/[*n*(*n *− 1)], where *n* represents the number of nodes. It is important to note that for the PPN networks investigated in this study, the number of edges were markedly smaller than the number of possible connections rendering the network density estimates small Pre-HB1 and Post-HB1. The S estimates (S ≫ 2) from surrogate testing revealed that the community structure of the PPN were markedly different from those random graph models rejecting these as potential generative mechanisms of PPN Pre-HB1 and Post-HB1, i.e. community structures of the PPN may not be sufficiently explained by synthetic random graph models investigated in the present study. However, random graphs did prove to be useful internal controls and comparing the PPN properties to their random graph surrogates may be preferred as opposed to a direct comparison of the PPNs at Pre-HB1 and Post-HB1 since the number of nodes and edges of the PPN are not the same at Pre-HB1 and Post-HB1. The S estimates at Pre-HB1 was relatively higher than that of Post-HB1 indicating that the strength of deviation of the community structure from the random graphs decreased Post-HB1. These essentially may reflect the effectiveness of House Bill 1 in reducing the patient movement patterns. From the results presented, understanding the patient movement patterns and objectively quantifying its changes using rigorous network-based approaches has the potential to assess the effectiveness of policy as well as enable targeted surveillance of prescriber communities.

There are several limitations to the present study. The cross-sectional data in this study was deliberately restricted to a single month, since the probability of patients seeking drugs of the same schedule from multiple prescribers within this time frame is expected to be minimal. However, incorporating explicit time stamps of the patient movement between prescribers may assist in determining the sequence of prescription patterns across different prescriber specialties and assess temporal evolution of PPNs. Incorporating explicit temporal information may also assist in predicting the patient drug seeking behavior ahead of time using approaches such as link prediction^29^ and assist in future policy interventions. It is also important to note that the geographical distances between the prescribers were not explicitly accommodated in the methodological approaches presented in this study. While we present compelling evidence of characteristic variations in statistical and network characteristics Pre-HB1 and Post-HB1, these may not necessarily imply a causal association between the observed changes and House Bill 1 implementation. The present study also assumes the PPN to be unweighted undirected graph but it is possible to assign weights to edges when a patient seeks multiple prescriptions between pairs of prescribers. While the results are presented in a Medicaid population in Kentucky, a more comprehensive study across distinct settings is required to establish the enhanced generalizability of the findings.
